# Complete chloroplast genome of glyphosate resistant *Conyza bonariensis* (L.) Cronquist from Australia

**DOI:** 10.1080/23802359.2017.1357441

**Published:** 2017-07-25

**Authors:** James P. Hereward, Jeff A. Werth, David F. Thornby, Michelle Keenan, Bhagirath Singh Chauhan, Gimme H. Walter

**Affiliations:** a School of Biological Sciences, University of Queensland, Brisbane, Australia;; b Queensland Department of Agriculture and Fisheries, Leslie Research Centre, Toowoomba, Australia;; c Innokas Intellectual Services, Coomera, Australia;; d The Centre for Plant Science, Queensland Alliance for Agriculture and Food Innovation (QAAFI), University of Queensland, Gatton, Australia

**Keywords:** *Conyza bonariensis*, fleabane, glyphosate resistance, weed

## Abstract

*Conyza bonariensis*, flaxleaf fleabane, is a serious weed in Australian agricultural systems, particularly the north-east cropping system. We present the complete chloroplast sequence of *C. bonariensis* reconstructed from Illumina whole genome shotgun sequencing. This is the first complete chloroplast genome available for genus *Conyza*. The complete chloroplast sequence is 153,014 bp long, and has the same gene content and structure as other members of the tribe Astereae. A Bayesian phylogeny of the chloroplast coding regions of 18 representatives of Astereae is presented. The *C. bonariensis* chloroplast genome is deposited at GenBank under accession number MF276802.

*Conyza bonariensis* (L.) Cronquist (Asteraceae), also known as flaxleaf fleabane, is a serious weed of north-eastern cropping systems in Australia and glyphosate resistant populations were first collected there in 2006 (Walker et al. 2011). This species has evolved glyphosate resistance in at least nine different countries (Heap 2017) and several species in the genus have evolved glyphosate resistance. *Conyza bonariensis* is allohexaploid with a chromosome count of 2*n* = 6*x* = 54 (Thebaud and Abbott 1995; Paula and Pinto-Maglio 2015). Several species in this genus are polyploid but their ancestry and evolutionary relationships are not clear. No complete chloroplast sequences have been made available for this genus on public databases, despite the publication of the nuclear genome for *C. canadensis* (Peng et al. 2014).

We assembled the complete chloroplast of *C. bonariensis* from deep sequencing of a sample from the highly glyphosate resistant line Q17 (see Walker et al. 2011 for collection details, and a voucher is held by JPH at the University of Queensland). DNA was extracted from leaf material using CTAB followed by spin column purification. Genomic sequencing libraries were constructed using the NebNext Ultra DNA kit (New England Biolabs, Ipswich, MA) and a full lane of PE150 Illumina sequencing was conducted by Novogene (Beijing, China). The chloroplast sequence was assembled in Geneious v9.1.3 (http://www.geneious.com, Kearse et al. 2012) by mapping assemblies made with SOAPdenovo (Luo et al. 2012) to the *Helianthus giganteus* complete chloroplast sequence (Accession = NC_023107). This was followed by extension of the contigs using iterative read-mapping; these contigs were then ordered and joined by alignment to the reference. The final consensus sequences were checked manually to ensure correct mapping distances across the assembly. Read mapping and assembly alone were unable to close the chloroplast at the start position, so two primers were designed (F = GGAGGAAGCTGTGACACGTT, R = CTATTGAAGCTCCATCTACAAATGG) and sanger sequencing performed to finish the genome.

All complete chloroplast sequences for the tribe Astereae were downloaded from GenBank. One representative per genus was selected. The complete chloroplasts were aligned using MAFFT (Katoh and Standley 2013), and all non-coding sequences were removed from the alignment. The GTR + I+G model of nucleotide substitution was found to be the most likely by jmodeltest2 (Darriba et al. 2012). A Bayesian phylogenetic tree was produced using Mr. Bayes (Huelsenbeck and Ronquist 2001) under this substitution model with *Helianthus annuus* (CM007907) used as the outgroup (Figure 1). Annotations were made based on this alignment.

**Figure 1.**
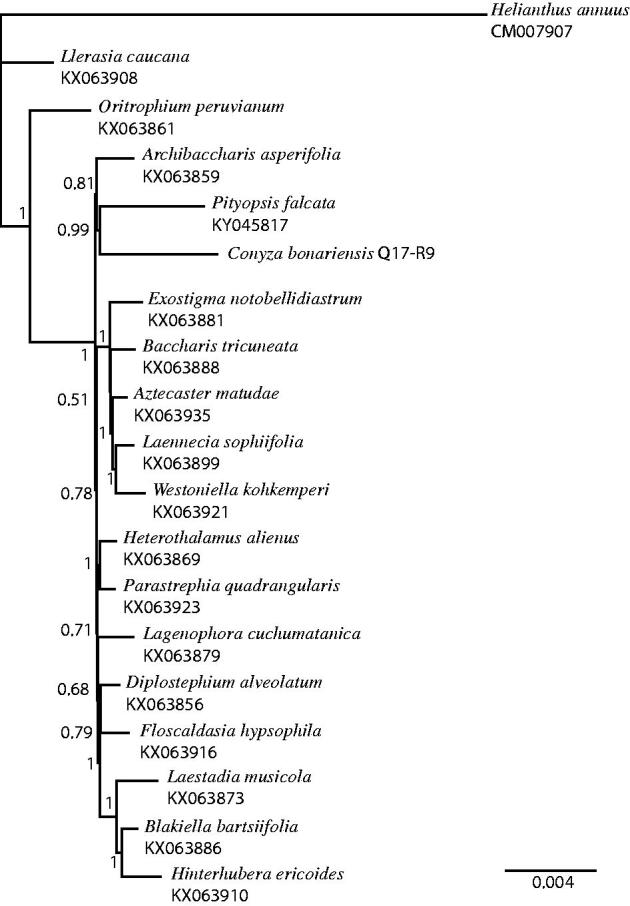
Phylogenetic tree produced using Bayesian estimation (Mr. Bayes) of complete chloroplast genomes from tribe Astereae, one representative per genus, with *Helianthus annuus* as the outgroup, node labels indicate the posterior probability after 1 × 10^6^ iterations.

The complete chloroplast sequence of *C. bonariensis* is 153,014 bp and the gene content, gene order and structure of the inverted repeats are similar to most members of the tribe. The phylogeny of tribe Astereae, based on the complete chloroplast coding sequences, places *C. bonariensis* closest to *Pityopsis*, but sequences are available for only 18 of 170 genera in this tribe (Noyes and Riesberg 1998). This complete chloroplast genome (GenBank accession MF276802) will allow for the identification of chloroplast reads from ongoing transcriptomic analysis of glyphosate resistance in this species.
